# HiView: an integrative genome browser to leverage Hi-C results for the interpretation of GWAS variants

**DOI:** 10.1186/s13104-016-1947-0

**Published:** 2016-03-11

**Authors:** Zheng Xu, Guosheng Zhang, Qing Duan, Shengjie Chai, Baqun Zhang, Cong Wu, Fulai Jin, Feng Yue, Yun Li, Ming Hu

**Affiliations:** Department of Biostatistics, University of North Carolina, Chapel Hill, NC 27599 USA; Department of Genetics, University of North Carolina, Chapel Hill, NC 27599 USA; Department of Computer Science, University of North Carolina, Chapel Hill, NC 27599 USA; Curriculum in Bioinformatics and Computational Biology, University of North Carolina, Chapel Hill, NC 27599 USA; School of Statistics, Renmin University of China, Beijing, 100872 China; College of Veterinary Medicine, Nanjing Agricultural University, Nanjing, 210095 Jiangsu China; Department of Genetics and Genome Sciences, Case Western Reserve University, Cleveland, OH 44106 USA; Department of Biochemistry and Molecular Biology, Institute for Personalized Medicine, Pennsylvania State University College of Medicine, Hershey, PA 17033 USA; Division of Biostatistics, Department of Population Health, New York University School of Medicine, New York, NY 10016 USA

**Keywords:** Integrative genome browser, Hi-C data, GWAS variants

## Abstract

**Background:**

Genome-wide association studies (GWAS) have identified thousands of genetic variants associated with complex traits and diseases. However, most of them are located in the non-protein coding regions, and therefore it is challenging to hypothesize the functions of these non-coding GWAS variants. Recent large efforts such as the ENCODE and Roadmap Epigenomics projects have predicted a large number of regulatory elements. However, the target genes of these regulatory elements remain largely unknown. Chromatin conformation capture based technologies such as Hi-C can directly measure the chromatin interactions and have generated an increasingly comprehensive catalog of the interactome between the distal regulatory elements and their potential target genes. Leveraging such information revealed by Hi-C holds the promise of elucidating the functions of genetic variants in human diseases.

**Results:**

In this work, we present HiView, the first integrative genome browser to leverage Hi-C results for the interpretation of GWAS variants. HiView is able to display Hi-C data and statistical evidence for chromatin interactions in genomic regions surrounding any given GWAS variant, enabling straightforward visualization and interpretation.

**Conclusions:**

We believe that as the first GWAS variants-centered Hi-C genome browser, HiView is a useful tool guiding post-GWAS functional genomics studies. HiView is freely accessible at: http://www.unc.edu/~yunmli/HiView.

**Electronic supplementary material:**

The online version of this article (doi:10.1186/s13104-016-1947-0) contains supplementary material, which is available to authorized users.

## Findings

The eukaryotic genome is organized at multiple levels ranging from chromosomal territories to topologically associated domains. Such hierarchical three-dimensional organization is closely related to genome function [1]. Historically, the study of genome organization has relied on microscopy-based techniques, which suffers from low resolution and low throughput. Recently, a series of technologies based on chromatin conformation capture (3C) [2], such as Hi-C [3] and in situ Hi-C [4], have been developed, enabling a high resolution genome-wide view of chromosomal architecture.

Data from 3C-based technologies can shed light on the structural and functional mechanisms, including non-coding variants identified for complex trait associations in genome-wide association studies (GWAS). GWAS has been resoundingly successful, identifying thousands of variants associated with complex traits. However, only a small proportion (7–12 %) of these variants fall into protein coding regions [5], making the interpretation of non-coding variants imperative. With the help of 3C-based technologies, a recent study [6] identified long-range (at megabase distances) interactions between the obesity-associated intronic variants in *FTO* gene and the promoter region of homeobox gene *IRX3*, demonstrating it is the expression of *IRX3* rather than *FTO* that is directly linked to body mass and composition. This study showcased the power of 3C-based technologies for elucidating the functional mechanisms of genetic variants implicated by GWAS.

As 3C-derived technologies have been increasingly widely used, multiple visualization tools have been devised recently, such as Hi-C data browser [3] and 3D genome browser [7]. In addition, WashU EpiGenome browser is widely utilized for simultaneous visualization of Hi-C and other epigenetic data from the Roadmap Epigenomics project [8]. Most recently, Juicebox has been developed for visualizing the in situ Hi-C data [4]. Meanwhile, HiBrowse [9] has been developed to facilitate statistical analysis of Hi-C data.

Although many useful visualization tools have been developed, none of them is able to display 3C-based data with a focus on GWAS variants interpretation, preventing researchers from fully mining rich information, generating testable hypothesis, and visually validating biological findings. In addition, few of them incorporates peak calling results from 3C-based data or shows the magnitude of statistical evidence, making the interpretation of the statistical significance of 3C-based data extremely challenging.

To fill in the above gaps, we present HiView, the first genome browser for GWAS-variant centered visualization of Hi-C data. Additional file 1: Figure S1 shows the user interface of HiView. Users can select and extract genomic annotation of a GWAS variant by selecting the marker type and specifying the marker name. HiView displays raw and expected count data, and measures of statistical significance from several state-of-the-art Hi-C peak callers, such as AFC [10], Fit-Hi-C [11] and a hidden Markov random field (HMRF) based Hi-C peak caller [12]. By creating an ensemble of peak calling results from different approaches, users can have more robust data interpretations. For gene annotation, HiView incorporates three gene annotation tracks: (1) Ensembl genes, (2) UCSC genes and (3) RefSeq genes.

**Fig. 1:**
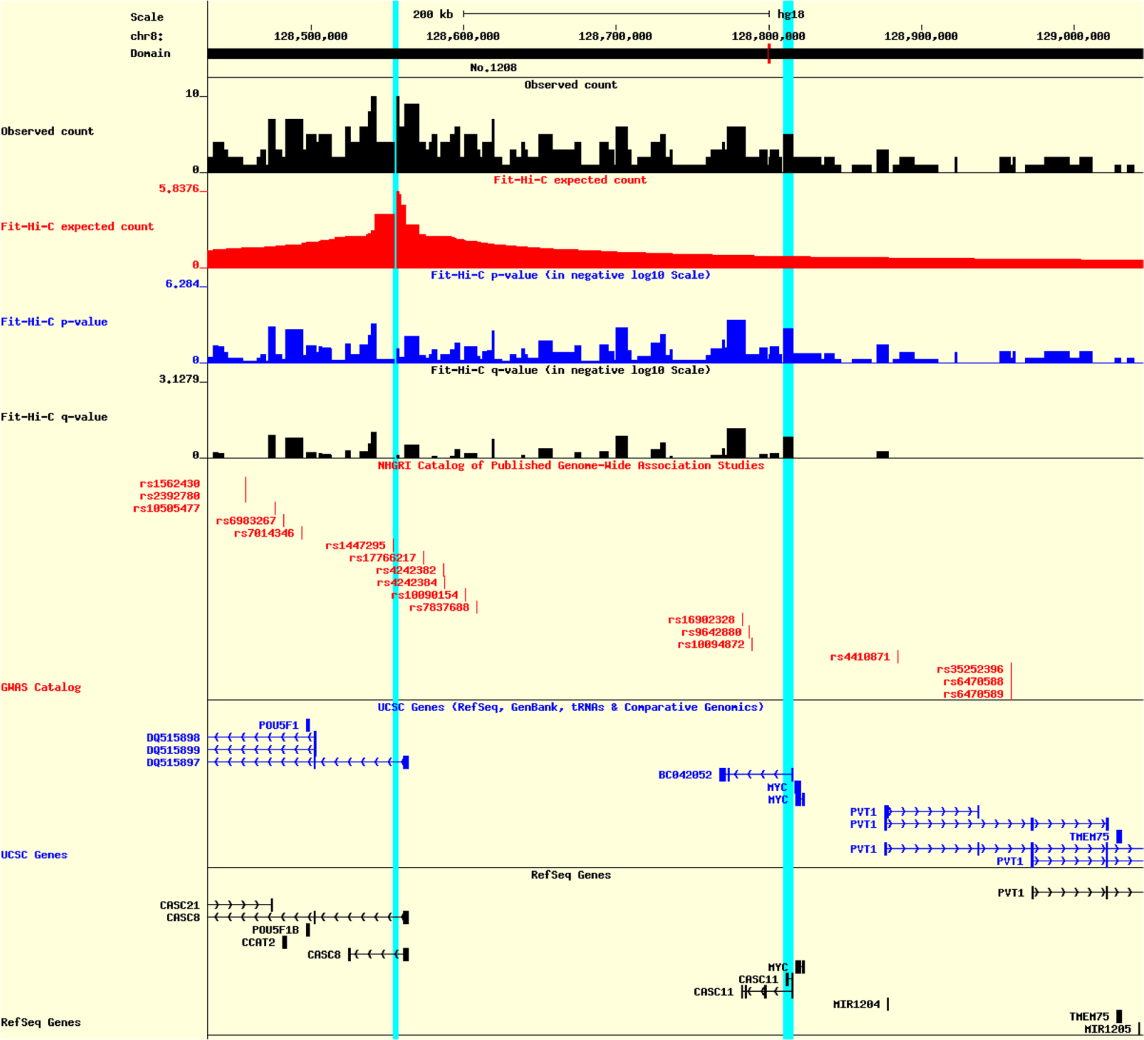
HiView snapshot of GWAS variant rs1447295. The *left* and *right* light *blue* bars highlight the location of GWAS variant rs1447295 and gene *MYC*, respectively. Using Hi-C data from human IMR90 cells, we observe five paired-end reads spanning between rs1447295 and the transcription start site of gene *MYC*, while the expected contact frequency is 0.8281. Such long-range chromatin interaction is statistically significant, with p-value 0.0016. Therefore, we hypothesize that gene *MYC* is a potential target of this likely regulatory GWAS variant rs1447295

Users can configure HiView for customized visualization in many ways (detailed in the online tutorial) including but not limited to (1) selecting tracks to display, (2) specifying the order of displayed tracks, (3) moving the viewing window upstream and downstream, zooming in and out, and specifying the range of the viewing window, (4) specifying the genomic regions to highlight, (5) specifying the text and color used for each track and (6) specifying the picture size and width. HiView also provides a table of numerical values of Hi-C data and peak calling results that can be downloaded by users. Figures 1 and Additional file 1: Figure S2 show an example of HiView figure and HiView table, respectively. A detailed tutorial to generate Fig. 1 can be found in the Additional file 1: Section S1.

Here is an example of using HiView to leverage Hi-C results for the interpretation of GWAS variants. Multiple studies [13, 14] have identified rs1447295 to be associated with the risk of prostate cancer. Although rs1447295 was mapped as an intronic variant in *CASC8* lncRNA, its functional mechanisms are still unknown. Both RgulomeDB [15] and HaploReg [16] identify this variant as an enhancer for multiple cell lines, indicating its potential regulatory role. Using the high resolution fragment level Hi-C data from human IMR90 lung fibroblastic cells [10], we observed statistically significant long-range chromatin interactions between rs1447295 and the transcription start site of the *MYC* gene with *p* value 0.0016 (Fig. 1). Therefore, we hypothesized that *MYC* gene is a potential target of this likely regulatory GWAS variant rs1447295 [17]. In this work, the Hi-C data and GWAS variant were collected from different cell types. It would be more informative to integrative Hi-C data and GWAS variants from the same cancer cell line, to fully understand the mechanistic relationship. As Hi-C data from more tissue and cell types are generated, we will have a more comprehensive understanding of tissue or cell type specific target genes.

The HiView interface is implemented using PHP, HTML and cascading styling sheets (CSS) languages. Hi-C and GWAS data are stored in a MySQL database in the UNC Linux server. HiView is compatible with Internet Explorer, Chrome and Firefox. HiView also allows users to upload their own Hi-C dataset for customized comparison and visualization.

In summary, we present HiView, a visualization tool that integrates raw Hi-C data and chromatin interactions identified by various peak callers for the interpretation of GWAS variants. HiView is the first genetic GWAS-variant centered visualization tool for Hi-C data. The resulting one-dimensional view allows close examination of interactions between each GWAS variant and all genes in the region the variant resides. We believe that HiView will facilitate the interpretation of GWAS variants, particularly the identification of their potential target genes.

## Availability and requirements

Project name: HiView.

Project home page: http://www.unc.edu/~yunmli/HiView.

Operating system(s): Platform independent.

Programming language: PHP, HTML and cascading styling sheets (CSS) languages.

Other requirements: browser such as Internet Explorer, Chrome and Firefox.

License: GNU GPL (version 3, 06/29/2007).

Any restriction to use by non-academics: none.

## Availability of supporting data

Original raw data used in Fig. 1, Additional file 1: Figures S1 and S2 were retrieved from the NCBI Gene Expression Omnibus repository (GSE43070: http://www.ncbi.nlm.nih.gov/geo/query/acc.cgi?acc=GSE43070).

## Additional file

10.1186/s13104-016-1947-0 HiView graphic user interface. Users can input the genomic location of a GWAS variant by (1) select the marker type, (2) type the marker name and then (3) click the Run button (red-colored). Users can configure HiView in many ways (refer to online tutorial) to obtain customized figures. HiView is freely accessible at http://www.unc.edu/~yunmli/HiView. Figure S2. HiView Table for GWAS variant rs1600249.
