# Supplementary material for: HiView: an integrative genome browser to leverage Hi-C results for the interpretation of GWAS variants
Source: BMC Res Notes. 2016 Mar 11;9:159. doi: 10.1186/s13104-016-1947-0 (PMC4788823; doi:10.1186/s13104-016-1947-0)
Supplement: Supplementary file 1 — 10.1186/s13104-016-1947-0 HiView graphic user interface. Users can input the genomic location of a GWAS variant by (1) select the marker type, (2) type the marker name and then (3) click the Run button (red-colored). Users can configure HiView in many ways (refer to online tutorial) to obtain customized figures. HiView is freely accessible at http://www.unc.edu/~yunmli/HiView. Figure S2. HiView Table for GWAS variant rs1600249. [file 13104_2016_1947_MOESM1_ESM.docx]

**HiView: an integrative genome browser to leverage Hi-C results for the interpretation of GWAS variants**

**Additional file 1**

**Figure S1. HiView graphic user interface.** Users can input the genomic location of a GWAS variant by (1) select the marker type, (2) type the marker name and then (3) click the Run button (red-colored). Users can configure HiView in many ways (refer to online tutorial) to obtain customized figures. HiView is freely accessible at <http://www.unc.edu/~yunmli/HiView>


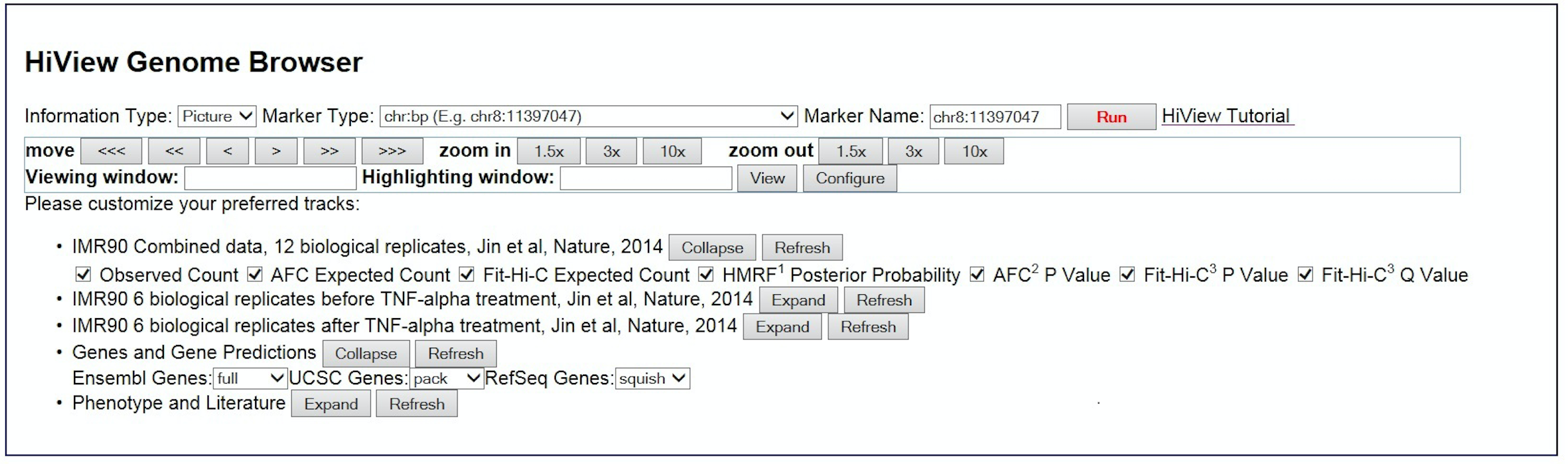


**Figure S2: HiView Table for GWAS variant rs1600249.**


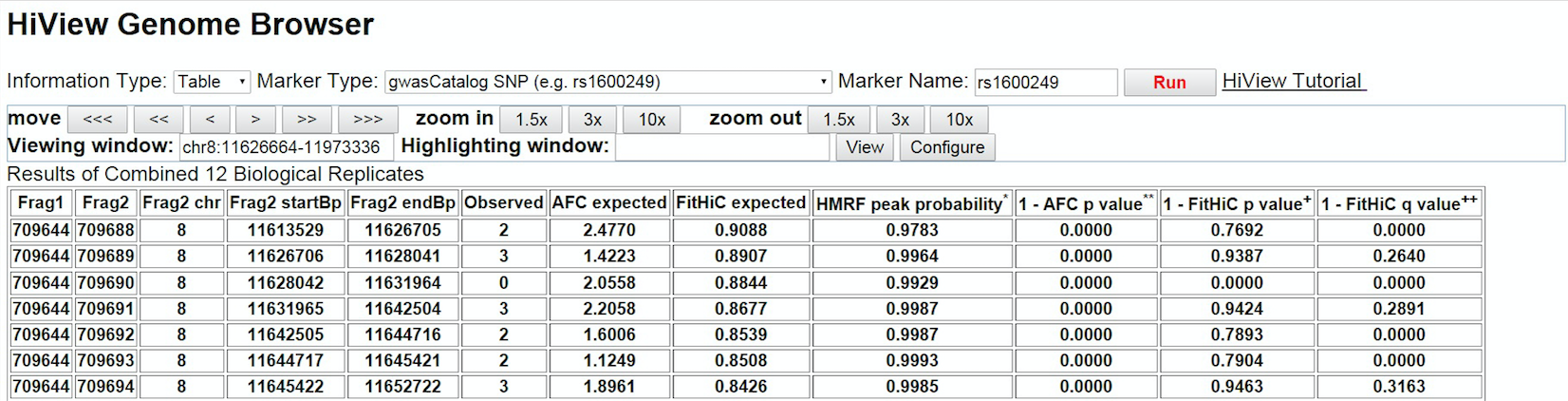


**Section S1. A detailed tutorial to generate Figure 1: HiView snapshot of GWAS variant rs1447295**

Users can generate “Figure 1: HiView snapshot of GWAS variant rs1447295” according to the following procedure:

1. Go to HiView homepage/mainpage, select information type as “Picture”, select “Marker Type” as “gwasCatalog SNP”, type “rs1447295” as the marker name, and then click the “Run” button to get a preliminary figure.
2. Specify the tracks to display. To generate the Figure 1 showed in the manuscript, use IMR90 combined data, and select “Observed count”, “Fit-Hi-C expected count”, “Fit-Hi-C p-value” and “Fit-Hi-C q-value”. In addition, set the display modes for Ensembl gene, UCSC genes and RefSeq genes to be “hide”, “pack” and “squish” respectively.
3. Configure the figure by user’s preference. To generate the Figure 1, users can click “Configure” button to go to the configuration webpage. In the configuration webpage, (1) Ensure all boxes, “Display left text”, “Display ruler”, “Highlight tag fragment”, “Display domain”, “Display domain number” and “Display isoform” are all checked (by default) (2) specify the sequence of tracks in the picture as “1,3,5,6,22,26,27”, and (3) specify the colors of specific tracks to be the same as the same color used in Figure 1 (by default, UCSC Genes are in green. Change this to blue to get a figure identical to Figure 1). Then click “Save” button to save the configuration and “View” button to go back to the homepage of HiView.
4. Navigate along the chromosome by zoom in, zoom out, moving and specify viewing windows to find genes and SNPs of interested. To generate Figure 1, user can type chr8:128433000-129041000 in the viewing window.
5. Highlight the fragment showing statistically significant long-range chromatin interaction with SNP rs1447295. To generate Figure 1, type in the highlighting window the range for the fragment chr8:128809372-128815174.
6. Click the “View” button to regenerate the figure. Right click the mouse and select “Save As” to download the generated figure.
